# Ten Simple Rules for Writing a PLOS Ten Simple Rules Article

**DOI:** 10.1371/journal.pcbi.1003858

**Published:** 2014-10-23

**Authors:** Harriet Dashnow, Andrew Lonsdale, Philip E. Bourne

**Affiliations:** 1 Bioinformatics, Murdoch Childrens Research Institute, Parkville, Victoria, Australia; 2 Life Science Computation Centre, Victorian Life Sciences Computation Initiative, Carlton, Victoria, Australia; 3 The University of Melbourne, Parkville, Victoria, Australia; 4 The Australian Research Council Centre of Excellence in Plant Cell Walls, School of Botany, University of Melbourne, Parkville, Victoria, Australia; 5 Office of the Director, National Institutes of Health, Bethesda, Maryland United States of America; Whitehead Institute, United States of America

*[PB: When I read the title of this article I laughed out loud—how many times has that happened to you when reading professional articles? Laughter is good whatever the context. When I started the series in 2005, I had no idea it would be so successful. This article, which I had no part in writing, only adding commentary shown in italics, is in my mind a celebration of that success. My commentary is simply to provide a historical perspective to explain some aspects of why the collection is the way it is and, of course, to make a few personal observations which, after all, is what the collection is meant for. Thanks to HD and AL for making this happen and for including me as an author (see Rule 4) and to all those that have contributed over the past nine years.]*

## Introduction

Would Newton retweet your post on Twitter? How would Einstein view open source software? How would Darwin have handled a Wikipedia edit war?

The way we do science is changing almost as fast as the volume of our data. Advice is needed; however, advice on leading a successful scientific life is usually confined to outdated memoirs, unrecorded weekly lab meetings, neglected blogs, or casual conversations at a conference.

When we are faced with the challenges of how to be the best scientist we can, our instinctive reaction is to follow our usual pattern of inquiry—search the literature.

This search left us wanting, until we discovered the PLOS Ten Simple Rules collection. We have found them to be a series of concise articles that capture the professional zeitgeist of being a scientist in an approachable manner.

Many topics cover the professional (or “soft”) skills that are necessary for a modern scientific career, but are not part of a formal scientific education. *[PB: Sad but true—teaching such skills should be a no-brainer.]* These articles represent an invaluable chance to pass on advice and knowledge in a way that can be widely distributed, formally recognised, and—as an added benefit—cited.

If (like us) you have read some articles in the Ten Simple Rules collection and appreciated their value, you may feel the urge to write one of your own. The collection provides a succinct and engaging format for advice on these skills. However, coming up with an article on soft skills need not be hard. Perhaps you have some insight, experience, or wisdom to impart. How would you do that?

Is there practical advice for contributing to the Ten Simple Rules collection already available? What can we learn from the existing articles in the collection? If only there was an article with ten simple rules for writing a PLOS Ten Simple Rules article. If only that article could be peppered with insightful comments from the founder of the collection: Philip E. Bourne.

This is that article.

## Rule 1: Have Ten Rules

**Figure 1:**
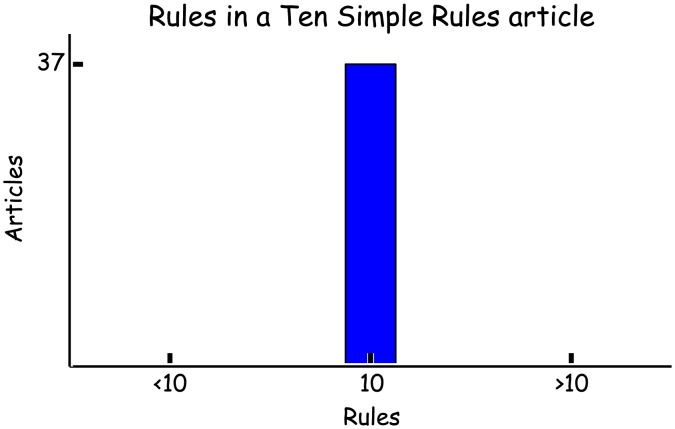
Have ten rules.

Perhaps the most obvious prerequisite for writing an article entitled “Ten Simple Rules…” is to actually have ten rules (Figure 1). There can be a temptation to include unimportant points or to split one topic over multiple rules to get to that magic number ten. If you can't think of ten rules, maybe your topic is too specific for this format.

Another common problem is having too much to talk about. This one is a little easier to deal with. Don't be tempted to emulate Spinal Tap by “turning it up to eleven.” Simply rank your ideas by how important or how thought provoking they are and then just write about the top ten. Alternatively, if you have twenty rules and there is a clear split, you may have two articles on your hands. There may be scope to combine a few related ideas within one rule, but don't get carried away. Your Ten Simple Rules should be just that, simple.

*[PB: Surprisingly, in the Ten Simple Rules articles I have written, I have found the imposition of ten never to be an issue—somehow it has seemed just enough, but not too much. Undoubtedly important points have been omitted but what is there seems to hang together. When authors suggest a topic for the Ten Rules I say send the Rules first no text. If they stand alone and say something new then I encourage them to flesh them out.]*

## Rule 2: Choose Your Topic Wisely

The articles in the Ten Simple Rules collection share an almost intangible common component: everything you always wanted to know about science (but perhaps were afraid to ask). These are articles about how to get by in the world of scientific research. Some give specific guidance in the field of bioinformatics and computational biology (naturally, as the collection originated in *PLOS Computational Biology*), but most offer broad advice that reaches far beyond this demographic.

Consider the topics covered so far: advice to graduate students, getting the right postdoctoral position, choosing between academia and industry, and how to start a company. The articles even extend to interpersonal skills like networking, collaboration, and communicating with your supervisor. There is also a strong academic focus: the core skills of doing research, writing, publishing, and teaching.

Now, what can you add to the mix? What do you wish that you'd known a year ago? What would you tell a new student? What do you wish your supervisor would realise? Computational biology is such a fresh, fast-moving field. There is always room for advice to emerge and for those new to the field to share their experiences, drawing from other disciplines and bringing together emerging ideas and techniques. The Ten Simple Rules collection is a reservoir for accessible wisdom (in both the open source and intellectually approachable senses of the word). What do you have to contribute?

If you are stumped, here are some suggestions: Ten Simple Rules for retiring when you know you should. Ten Simple Rules for winning a Nobel Prize. Ten Simple Rules for managing a scientific rivalry.

*[PB: Some would say I should write the first; none would say I should write the second; the third I have enough of already, but having an entry in the series from a Nobel laureate is a great idea—I am working on recruiting that someone right now—and so the series goes.]*

## Rule 3: Include an Introduction

Never underestimate the importance of a great introduction. Your introduction defines the scope of your article. It sets up a promise to your reader that you will cover this, that, but not some other thing. It provides the opportunity to put your topic in perspective and fill any gaps in your reader's knowledge.

Perhaps the most vital function of the introduction is to convince your reader to keep reading. You need to make your point clearly and simply the first time. You are also going to need to catch their attention in the first paragraph or so. You must convince them that you can both entertain and inform.

Like your Ten Simple Rules, your introduction should be short and to the point—there is no point having Ten Simple Rules with a thesis-length introduction.

*[PB: Agreed, particularly concerning the length of the introduction.]*

## Rule 4: Be Philip E. Bourne

**Figure 2:**
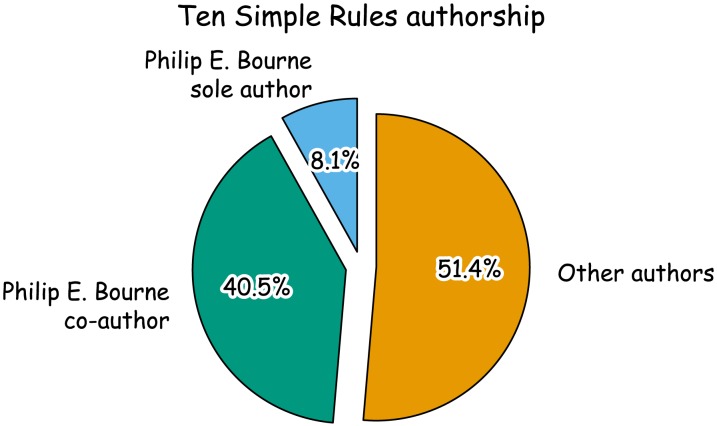
Your best chance of having a PLOS Ten Simple Rules article published is by being Philip E. Bourne.

Your best chance of having a PLOS Ten Simple Rules article published is by being Philip E. Bourne. At the time of writing, 48.6% of PLOS Ten Simple Rules articles list *PLOS Computational Biology* Founding Editor-in-Chief, Philip E. Bourne, as the first, last, or sole author (Figure 2). In fact, statistically speaking (Box 1), his frequent occurrences as an author of Ten Simple Rules is significant (p-value = 6.63758e-52).

**Box 1. Philip E. Bourne Is Significantly Over-Represented as an Author in the PLOS Ten Simple Rules Collection**

Showing the methods in the main text for the statistical significance of being Philip E. Bourne went against the flow of our article, and might have scared readers off. Therefore we've hidden them here to show that even in a Ten Simple Rules article, you need to back up your statistical claims.

We consider Bourne as an author in the Ten Simple Rules collection through over-representation analysis, where we look for over-representation of authorship in our group of interest, the Ten Simple Rules collection, compared to the overall PLOS collection.

Let: k = Number of Ten Simple Rules articles where Bourne is an author, n = Total number of Ten Simple Rules articles, K = Number of PLOS articles where Bourne is an author, and N = Total number of PLOS articles.

Then Pr(X = k)∼hypergeometric.

k = 18, n = 37, K = 55, N = 119435

Pr(x> = k) = phyper(k-1, K, N-K, n, lower.tail = FALSE) # R code

 = 6.63758e-52

Bourne is significantly over-represented as an author in the Ten Simple Rules collection (compared to what we would expect by chance based on his publication rate in the entire PLOS archive).

We speculate that as the Founding Editor-in-Chief of *PLOS Computational Biology* and founder of the series, Bourne is positively disposed towards publishing articles in the collection, and this disposition accounts for the high number of Ten Simple Rules articles he has authored.

Further experiments are required to validate this theory. Unfortunately, ethics approval to experiment on Bourne was not forthcoming. Bourne himself registered strong objections to our proposed “knock out” tests.

*[PB: He has been knocked around enough already over his career—wait that could be a topic for a new Ten Simple Rules article.]*

More recent articles are increasingly written by other experts or groups of individuals, but Philip is often found as part of the author list. Before you start filling out a change of name form though, consider asking Philip to be a coauthor.

*[PB: And I thought they asked me because I know something.]*

## Rule 5: Collaborate

You have a great idea for a Ten Simple Rules article, now you need to make it a reality. We have discussed getting Philip Bourne involved (see Rule 4), but we haven't yet discussed why having a coauthor or two could help, even if they don't have an in at PLOS.

When you write a Ten Simple Rules article, you are speaking with an aura of knowledge and authority. One way to achieve this is to be a giant in your field. But if only the “experts” write these articles, we are missing out on the intimate, hard-earned knowledge of those in the trenches. If your name doesn't strike awe in your readers, perhaps consider combining forces with others. If you can't find experts, contact your peers. The emerging opinion of a group of more junior authors may be almost as respectable, particularly if the topic at hand is relevant.

Crowdsourcing your peers is also a great way to find the right ten rules. Gather rules from a number of people and look at the intersection. This may give you a sense of the community consensus rather than an individual opinion.

*[PB: Agree that everyone has something to offer the collection. With respect to having “an in at PLOS,” I should say that all contributions for which I am not an author have me as an editor and are sent for review. Another of the senior editors handles those that have me as an author. Either way, they often require significant editing and, on occasion, are rejected.]*

## Rule 6: Research

When writing any academic paper, reading the literature is a given. So, read the Ten Simple Rules collection. There is no excuse: there are only ten rules per paper!

After you have read this article, and some of the other articles in the collection, you will have a good idea of the required style and tone.

To show that we can take our own advice, we first conducted a thorough review of the Ten Simple Rules, culminating in the first draft of this article.

To gain a more historical perspective we also searched the literature for titles containing “ten simple rules.”

The first entry in the PLOS Ten Simple Rules collection was published in 2005. The earliest entry matching our search was published in 1988 (“Ten simple rules for improving advertising in health care institutions”) and though the colloquial origin of the phrasing is beyond the scope of this paper, it is the first example we can find of a journal article that fits the format later used by the PLOS collection.

Articles within the PLOS collection have inspired articles in other fields, including blog posts, contributions to pre-print servers, and journals such as *NeuroImage* and the *International Journal for Parasitology: Parasites and Wildlife*.

The *NeuroImage* branch of ten simple rules articles, with a length, focus, and complexity that may call into question the use of the term “simple,” demonstrates how the ten simple rules format can be adapted to suit any need and discipline. The authors of this review resisted the urge to produce a phylogenetic tree. Just.

*[PB: I certainly make no claim to be the originator of the phrase “Ten Simple Rules.” It just seemed to fit the length and form of what was required to get the point across in that very first article, and clearly others thought the same as additions to the series came rolling in. The rumour that the appeal of “simple” is that each entry in the series is the right length for a bathroom or lavatory break should be disregarded.]*

## Rule 7: Write Well

The topic of writing well deserves its own ten simple rules. There are plenty of great online resources about how to write well. We won't try to enumerate the rules here, but instead will focus on one of the most important: know your audience.

So, who reads *PLOS Computational Biology*? Who reads the Ten Simple Rules articles specifically?

The audience is highly educated and has a great deal of specialist expertise. They may be skimming this article over a coffee in the middle of checking their email in the morning. Unlike a scientific article, the reader of a Ten Simple Rules article is unlikely to read it twice for comprehension, or to try to decipher any complex language.

There is no single writing style that you can successfully apply to every domain. To select your style, you must first know who you are writing for. Who is your audience? What are they interested in? What do they already know about this topic? How much time are they likely to spend reading this article?

If you can answer these questions, you are a long way towards getting the tone and content right.

*[PB: Wise words that in one way or another are stated in many of the articles in the series and so it must be true. In terms of style we have been flexible—if the style is different, but works for the content, so be it. This article is, of course, a case in point.]*

## Rule 8: Reference

The ten rules format calls for a casual writing style, but that shouldn't prevent you from referencing. The need for citations will vary with the topic, however this is still a scientific journal, and this paper will form part of the scientific literature, indexed and found. There is plenty of scope for presenting your opinion, but do back up your facts. Wherever possible cite relevant online resources, including other Ten Simple Rules articles.

The caveat to this rule is that there can be such a thing as too many references. A great Ten Simple Rules article is easy to read and accessible to its audience. There is little point in having ten simple rules then overloading them with hundreds of references. Include only those that you have read and learnt from.

**Figure 3:**
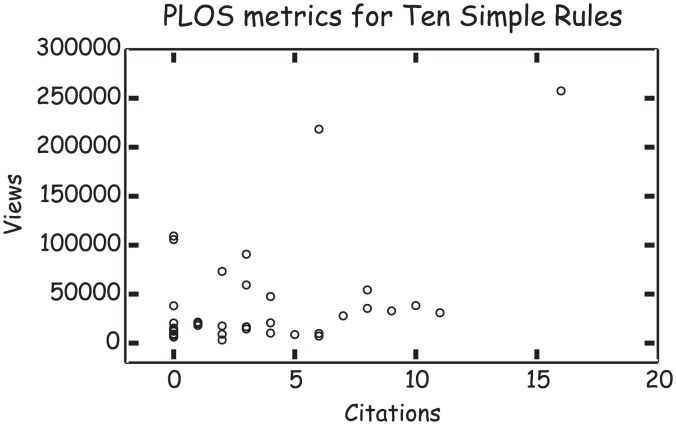
Ten Simple Rules articles are viewed more often than they are cited.

Resolve this conflict by considering how your article will be used. How many people will read it? Although references are important, Ten Simple Rules articles are viewed and downloaded more often than they are cited (Figure 3). In fact, by recent count, six of the top ten most viewed articles in *PLOS Computational Biology* were from the Ten Simple Rules collection. Clearly, the impact of your article will not be measured by citations.

By considering your audience (see Rule 7), you will get a sense for the number of citations needed to effectively get your message across. Balance accessibility, brevity and authority, as these qualities that will determine how many people have a chance to follow your ten simple rules.

*[PB: I love this graph (* *Figure 3* *). What does it say about impact? After all is this not what we are trying to measure? PLOS's efforts with article level metrics (ALMs) speak to the need to be more quantitative in how we measure the impact of a piece of scholarship. But since what we do most of the time is be qualitative—judging a piece of work by the impact factor of the whole journal—let me also be quantitative. Judging by the number of times someone I do not know comes up to me and says, “I know your work” and I respond, “really which research are you referring to?” Upon which they say, “I don't know your research, I am talking about the Ten Simple Rules,” I would say the impact is high—at least relative to my research.]*

## Rule 9: Edit

Once you have the words on the page, your next step is to edit them. Present your rules in a logical order. Order and reorder them. Find the flow both between and within your rules.

Once you have taken care of the big picture, spend some time thinking about whether your paragraphs are cohesive and well structured. This is not an essay, so the rules are a little more fluid, but you could still benefit from topic sentences and phrases that flow between paragraphs. If, like many academics, you find your sentences blow out beyond 30 words, you may like to use a tool such as Draft to keep them in check. These tools are great for picking up tortured sentence structures like passive voice and split infinitives.

One of the best ways to make your article more readable is to have others read it. Workshop and crowdsource your article. Your peers are your secret weapons. Many of them are your target audience, so you can do a test run and perhaps even get honest feedback.

Some literary theorists argue that as soon as you publish a piece of writing, it becomes the domain of the reader. The intent of the author pales in comparison to the interpretation of the audience. In this context, the reader is always right. So you may as well get the major criticisms and revisions out of the way early.

*[PB: As I say somewhere in the collection, what you commit to the literature will be around long after you are gone—it is a large part of your professional legacy—get it right. Reviewers' comments often relate to the order and organization of the rules, so this rule is good advice for getting your contribution published and out there.]*

## Rule 10: Have a Voice

Inject your personality. The coauthors of this article have included several jokes that did not make it past the editing stage. There was a joke about “soft skills informatics” and “professional development-omics” that was rightly cut, but that is part of the process. There are few scientific articles where you can borrow your plot style from the XKCD comic series (http://xkcd.com/), and this article is one of them.

That said, avoid in-jokes. Although perhaps incredibly funny to you, they don't tend to translate well to a wider audience. Your jokes should make most of your readers feel like they are part of the inner circle.

Does your voice match your article? In this article we have strived to balance humour with useful advice.

Our voice has mixed facts with seemingly irrelevant plots and calculations, and has attempted to convey a clear affection for the Ten Simple Rules collection, and shared useful tips to inspire you to write your own Ten Simple Rules.

*[PB: Personally I think it a shame that scientific discourse is taken more seriously the more impersonal it is. Why should humour and individuality impact the perceived value of science? With the Ten Simple Rules, you have the opportunity to buck that system as this article so rightly illustrates.]*

## Conclusion

You should definitely have a conclusion. Many readers will read the introduction, Rule 1, and then (if you're lucky) skim until they reach the conclusion. This is your opportunity to present a take-home message to your readers. Something to make sense of the rambling mess that is your article, despite extensive editing. Something to make it seem focused and insightful. It's also a great opportunity to re-inject your personality into your writing.

So here is our conclusion:

Congratulations on making it this far!

Hopefully we've convinced you that the Ten Simple Rules collection has a vital role to play in modern science. We'd like you to think about contributing to the discussion.

When you do, take care to choose your topic wisely. Success in this genre is all about knowing your audience. Decipher what they are interested in and how they interact with these articles. A great way of keeping your reader involved is to use your voice, play up your persona, and, of course, don't forget to have ten rules.

The rules listed in this article are a guide to forming your own ten simple rules. These rules are simple, but not trivial. Use this article as a guide, and get started. Today. Just take it one rule at a time.

@Newton, can I get a RT?

*[PB: I would like to think the articles I have written come from the heart, a genuine desire to short-circuit all the mistakes I have made in a long career. I think that desire to share your experiences so others can learn should be what guides you in writing a Ten Simple Rules article. I look forward to continuing to read your efforts.]*

## Supporting Information

Text S1 Data and code used to produce figures.
(DOCX)

