# Supplementary material for: Ten Simple Rules for Writing a PLOS Ten Simple Rules Article
Source: PLoS Comput Biol. 2014 Oct 23;10(10):e1003858. doi: 10.1371/journal.pcbi.1003858 (PMC4207461; doi:10.1371/journal.pcbi.1003858)
Supplement: Text S1 — Data and code used to produce figures. (DOCX) [file pcbi.1003858.s001.docx]

Figures 1, 2 and 3 were produced using the PLOS article level metrics API (<http://api.plos.org/>) and Python scripts available at: <https://github.com/lonsbio/10_simple_rules_analysis>

The top ten most viewed articles were calculated by advanced search on the PLOS Computational Biology website (<http://www.ploscompbiol.org/>) for all articles using a wildcard and then sorted by ‘Most views, all time’:

3,496 results for title:*. Accessed 12th August 2014.

Over-representation data from <http://almreports.plos.org/> accessed 20th June 2014, with the following searches:

18 results for (((title:"ten simple rules") NOT article_type:correction) NOT article_type:Correspondence) AND author:"Philip E. Bourne"; journals: All Journals

37 results for ((title:"ten simple rules") NOT article_type:correction) NOT article_type:Correspondence; journals: All Journals

55 results for NOT article_type:correction NOT article_type:Correspondence AND author:"Philip E. Bourne"; journals: All Journals

119,435 results for NOT article_type:correction NOT article_type:Correspondence ; journals: All Journals

*[PB: Does this mean I should publish more research articles?].*
